# Advancing real‐world data integration in nursing research: A policy perspective on Japan's next‐generation medical infrastructure law and the EU EHDS framework

**DOI:** 10.1111/jjns.70037

**Published:** 2025-12-22

**Authors:** Kazumi Kubota, Saeko Ishibashi

**Affiliations:** ^1^ Research Organization Shimonoseki City University Shimonoseki Japan; ^2^ Department of Healthcare Information Management The University of Tokyo Hospital Tokyo Japan; ^3^ Health and Global Policy Institute Tokyo Japan; ^4^ Department of Biostatistics, Graduate School of Medicine Yokohama City University Yokohama Japan; ^5^ School of Nursing Sapporo City University Sapporo Hokkaido Japan

**Keywords:** data governance, EHDS, electronic health records, Japan, nursing research, policy analysis

## Abstract

**Aim:**

This Viewpoint analyzes the policy implications of Real‐World Data (RWD) utilization in Japanese nursing research, comparing Japan's revised Next‐Generation Medical Infrastructure Law with the European Union's European Health Data Space (EHDS) regulation. The paper explores how data standardization, interoperability, privacy protection, and technological integration can be improved in Japan and proposes actionable policy solutions for RWD integration into nursing practice and health policy.

**Methods:**

The paper draws on a review of empirical studies and policy documents, comparing Japan's fragmented RWD framework with the EU's EHDS regulation. Focus areas include data standardization, interoperability, privacy protection, and technological integration. The review analyzes case studies from chronic disease management, nursing workflow optimization, and predictive analytics.

**Results:**

The comparison reveals barriers to RWD integration in Japan, such as inconsistent data standards, fragmented IT infrastructure, and lack of centralized data repositories. The EU's EHDS law offers a cohesive framework for cross‐border data sharing through standardized data formats, enhanced interoperability, and privacy protections. The paper argues that Japan must adopt similar policies to improve data governance, enhance interoperability, and develop integrated systems for nursing research.

**Conclusions:**

Japan must strengthen its data governance frameworks, establish centralized data repositories, and adopt standardized practices to fully capitalize on RWD's potential. By aligning its policies with the EU's EHDS regulation, Japan can improve RWD integration in nursing practice and health policy, leading to better outcomes and more effective interventions.

## 1 INTRODUCTION

Real‐World Data (RWD) has become a pivotal component in evidence‐based nursing practice, offering essential insights into clinical outcomes, resource allocation, and patient care management. The integration of RWD, particularly data from Electronic Health Records (EHR) and various public and private healthcare databases, facilitates the transition from a reactive to a proactive model in healthcare. It supports the management of chronic diseases, aids predictive modeling, and enhances clinical decision‐making. In nursing research, RWD plays an essential role in identifying best practices, optimizing workflows, and guiding evidence‐based policy decisions.

However, despite the significant potential of RWD, its effective integration into nursing research and practice remains a challenge, particularly in Japan. Japan's healthcare data infrastructure is fragmented, with numerous institutions using inconsistent data collection practices, varied coding methods, and different standards for data entry. This disjointed approach results in fragmented datasets, making it difficult to conduct large‐scale, cross‐institutional studies. Such fragmentation severely limits Japan's ability to generate reliable, high‐quality evidence to influence healthcare practices and policymaking.

Miyagawa et al. (2024) and Sugiyama et al. (2024) emphasize that these inconsistencies hinder the potential of RWD in Japan, complicating the development of a cohesive, data‐driven framework. Japan's fragmented data systems also impede efforts to improve clinical outcomes and policy reforms. In response to these challenges, the Japanese government revised the Next‐Generation Medical Infrastructure Law, aiming to integrate diverse healthcare data sources by linking EHR systems with public and private healthcare databases. This revision, as described by Yamamoto (2022), is a significant step toward addressing data fragmentation. It aims to create a more unified and accessible healthcare data system. However, despite the revision, Japan continues to struggle with standardizing data formats and ensuring data interoperability across institutions.

In addition to legislative efforts, the Science Council of Japan (SCJ, 2023) has highlighted the need to accelerate digital transformation in nursing. Its 2023 report, *Sustainable Nursing Digital Transformation Contributing to a Sustainable Society*, calls for standardized and interoperable nursing data systems, stronger data linkage across institutions, and the development of nursing professionals with informatics expertise, alongside clear regulations to ensure safe and ethical use of digital technologies.

In contrast, the European Union (EU) has successfully established a unified framework for RWD integration through its European Health Data Space (EHDS) regulation. The EHDS regulation mandates the use of uniform data formats, enhances data interoperability, and implements robust privacy protections, facilitating secure cross‐border data sharing (European Commission, 2025). The EU's approach has enabled multinational research and policy innovation, highlighting the importance of consistent, interoperable data systems to unlock the full potential of RWD. By adopting similar frameworks for data governance and integration, Japan can overcome its current barriers to RWD utilization and make more effective use of RWD in nursing research and healthcare policy.

This paper critically examines Japan's existing data integration framework, compares it with the EU's EHDS regulation, and proposes strategic policy solutions to enhance RWD utilization in nursing research, clinical practice, and health policy.

## 2 METHODS

This analysis is based on a narrative review of empirical studies, case studies, and policy documents from both Japan and the European Union. Sources were identified through targeted searches of PubMed, CiNii, and Google Scholar, as well as government and organizational websites, using combinations of keywords such as “real‐world data,” “nursing research,” “electronic health records,” “data governance,” “Japan,” and “European Health Data Space.” The search covered publications from 2015 to 2025, with a focus on peer‐reviewed journal articles, official policy documents, and representative case studies in nursing contexts. Selection was guided by relevance to the integration and governance of RWD in nursing practice and policy. Empirical studies were included if they addressed nursing workflows, chronic disease management, or advanced analytics using health data; policy documents were included if they outlined frameworks, laws, or strategies for RWD use.

The reviewed studies span various aspects of healthcare, including chronic disease management, nursing workflow optimization, and healthcare data governance. Watanabe et al. (2022) demonstrated the long‐term effectiveness of a disease management program aimed at preventing diabetic nephropathy using administrative data, emphasizing how RWD can improve chronic disease care. Sugiyama et al. (2024) examined integrated EHR systems in breast cancer care, illustrating how multidisciplinary healthcare teams, supported by EHR systems, can enhance patient outcomes by improving the accessibility and management of patient data. Takami et al. (2021) focused on nursing workflow optimization by analyzing gaze patterns during EHR data retrieval, providing insight into how RWD can enhance nursing practices and decision‐making.

In addition to these empirical studies, the review includes key policy documents influencing RWD usage in Japan and the EU. The European Commission's 2023 EHDS regulation mandates the use of uniform data formats, enhances data interoperability, and ensures strong privacy protections for data sharing across borders (European Commission, 2025). In Japan, the 2024 revisions to the Next‐Generation Medical Infrastructure Law aim to integrate EHR systems with public and private healthcare databases, seeking to streamline data flow between healthcare institutions (Japan Association for Medical Informatics, 2024). Despite these efforts, Japan faces significant challenges related to data standardization, inconsistent data entry practices, and fragmented IT infrastructure.

The review compares the data integration frameworks of Japan and the EU, highlighting differences in data governance and their impact on RWD utilization for nursing research, clinical practice, and policy development. This analysis also incorporates studies on advanced analytics, such as machine learning and natural language processing, to evaluate how emerging technologies are being integrated into RWD research to improve predictive modeling and decision‐making (Nakagami et al., 2021; Morioka et al., 2017; Morita et al., 2017).

## 3 RESULTS

The comparison between Japan's fragmented healthcare data systems and the EU's cohesive EHDS regulation reveals several critical gaps in RWD integration. Watanabe et al. (2022) and Sugiyama et al. (2024) emphasized the negative impact of inconsistent data standards and fragmented coding practices in Japan. These challenges hinder large‐scale, cross‐institutional studies, making it difficult to generate reliable evidence for clinical and policy decisions. Watanabe et al. (2022) highlighted that fragmented data prevent effective monitoring and evaluation of chronic disease management programs, complicating the creation of comprehensive healthcare strategies.

Takami et al. (2021) also pointed out that nursing workflows contribute to data discrepancies, particularly in the way nurses interact with EHR systems. These discrepancies, combined with inconsistent data entry practices, hinder the integration of RWD into clinical practice. Tomita et al. (2017) noted that such discrepancies often lead to adverse events, emphasizing the need for standardized data protocols across healthcare institutions. These findings underscore the importance of implementing consistent data standards and protocols for data entry to improve the effectiveness of RWD in nursing research.

In contrast, the EU's EHDS regulation mandates standardized data formats and ensures interoperability between member states, facilitating seamless data exchange. The regulation provides a secure framework for cross‐border data sharing, supporting multinational studies and the development of evidence‐based policies (European Commission, 2025). Real‐world implementations include FinnGen (2024), which integrates genomic and health data for precision medicine, and the TEHDAS joint action (TEHDAS, 2023), which develops governance and interoperability frameworks for the secondary use of health data across EU member states.

Furthermore, the EU demonstrates significant progress in nursing informatics under EHDS principles. Examples include remote patient monitoring systems integrated across multiple member states, AI‐assisted nursing workflows in Germany and the Netherlands, and standardized home care documentation initiatives such as the Swiss project (Kollmann et al., 2024). These developments illustrate the practical application of EHDS frameworks in supporting comprehensive nursing practice beyond institutional boundaries.

Nevertheless, while the EHDS is often presented as a strong model, the reality of implementation reveals significant hurdles: the current readiness across EU member states remains highly heterogeneous, and no single country is fully prepared to comply with the regulation (Kessissoglou et al., 2024). Additionally, scholars have raised concerns that the EHDS's scope may be overly ambitious—potentially “too big to succeed”—given the complexity of legal and regulatory alignment required (Marelli, 2023). Moreover, qualitative analysis points to governance challenges and barriers in implementing privacy‐enhancing technologies within the EHDS framework, highlighting a need for a strategic, integrated approach (van Drumpt et al., 2025).

Despite these challenges, EHDS still offers a more cohesive data ecosystem compared to Japan's fragmented system. However, Japan's fragmented IT infrastructure and lack of centralized data repositories continue to hinder the integration of healthcare data from various sources, limiting the effectiveness of RWD in improving clinical outcomes and informing policy decisions (Miyagawa et al., 2024; Tomita et al., 2017; Seto et al., 2018).

Table 1 summarizes the fundamental structural differences between the two frameworks, highlighting the specific areas where Japan must strengthen its approach to achieve effective RWD integration.

**TABLE 1:** Core Framework Differences Identified in This Study.

Framework element	Japan	EU EHDS
Data Scope	Anonymized provider‐generated data	Provider‐ and patient‐generated health data (EHR, PHR, wearable and other personal monitoring data)
Patient rights (PHR/wearable data)	No explicit legal rights to access, control, or portability of PHR or wearable data within this framework	Articles 3–7 guarantee individuals' rights to access, control, and portability of EHR, PHR, and wearable/monitoring data
Data standardization	Fragmented, inconsistent practices	Uniform data formats mandated
Interoperability	Limited interoperability across institutions; no cross‐border framework	Mandated cross‐institutional and cross‐border interoperability
Centralized infrastructure	Absent	Coordinated, multi‐country infrastructure framework established

The use of advanced analytics, including machine learning and natural language processing, has shown promise in enhancing the predictive and decision‐making capabilities of RWD. Nakagami et al. (2021) demonstrated how machine learning models can effectively predict in‐hospital pressure injuries, while explored the use of natural language processing to extract patient symptoms from clinical texts. However, both studies emphasized that the full potential of these technologies depends on the availability of high‐quality, standardized data, which is a significant challenge in Japan's fragmented data environment.

Collectively, these results indicate that Japan must adopt uniform data standards, establish centralized data repositories, and strengthen its data governance frameworks to improve RWD integration. By developing policies that address these issues, Japan can generate more reliable evidence and enhance data‐driven decision‐making in nursing practice and healthcare policy. In particular, implementing interoperable EHR systems and centralized repositories would enable the use of predictive analytics tools—such as those demonstrated for chronic disease management (Watanabe et al., 2022) and in‐hospital pressure injury prevention (Nakagami et al., 2021)—to support nurses in making timely, evidence‐based clinical decisions. Such infrastructure improvements would also create the foundation for a nationwide, data‐driven healthcare system that optimizes resource allocation, improves care coordination, and strengthens public health responses.

## 4 CONCLUSIONS

This analysis highlights the significant challenges Japan faces in integrating RWD into nursing research and healthcare policymaking. The fragmented nature of Japan's healthcare data systems, coupled with inconsistent data collection practices and privacy concerns, limits the effective utilization of RWD. Despite efforts to address these challenges through the revision of the Next‐Generation Medical Infrastructure Law, Japan continues to struggle with data standardization, interoperability, and the lack of centralized data repositories.

In contrast, the EU's EHDS regulation provides a robust model for RWD integration. The regulation mandates standardized data formats, enhances data interoperability, and ensures privacy protections, facilitating secure data exchange across borders. The EU's success in creating a cohesive and interoperable data ecosystem illustrates the benefits of standardizing data systems, and Japan can benefit from adopting similar frameworks to improve its own RWD infrastructure.

Japan must prioritize several key areas to fully unlock the potential of RWD. First, the development of uniform data standards across healthcare institutions is critical. Second, Japan should invest in creating centralized data repositories that facilitate large‐scale data sharing and research. Third, enhancing data governance frameworks and ensuring strong privacy protections are essential to building trust and enabling secure data sharing. By aligning its data policies with international best practices, particularly those demonstrated by the EU, Japan can overcome current barriers and fully leverage RWD to improve clinical outcomes, resource allocation, and healthcare policy.

Integrating standardized RWD within interoperable data systems will empower nurses to make more data‐driven decisions, optimize resource allocation, and ultimately improve patient care. By aligning with international standards in RWD governance, Japan can transform its healthcare system, leading to more efficient, evidence‐based healthcare solutions and better health outcomes.

## AUTHOR CONTRIBUTIONS

*Conceptualization and methodology*: Kazumi Kubota. *Data collection*: Kazumi Kubota and Saeko Ishibashi. *Data curation*: Kazumi Kubota. *Project administration*: Kazumi Kubota. *Resources*: Kazumi Kubota. *Manuscript writing (draft)*: Kazumi Kubota. *Manuscript writing (revised)*: Saeko Ishibashi and Kazumi Kubota. Kazumi Kubota and Saeko Ishibashi contributed equally to this work.

## CONFLICT OF INTEREST STATEMENT

The authors declare no conflicts of interest.

## ETHICS STATEMENT

Institutional Review Board (IRB) approval is not applicable as this manuscript is a Viewpoint paper and does not involve research on human subjects.
